# Supplementary material for: Angelica sinensis polysaccharide as potential protectants against recurrent spontaneous abortion: focus on autophagy regulation
Source: Front Med (Lausanne). 2025 Jan 15;12:1522503. doi: 10.3389/fmed.2025.1522503 (PMC11774876; doi:10.3389/fmed.2025.1522503)
Supplement: Supplementary material S1 — The main instruments used during the LC-MS process, along with their models/specifications and manufacturers. [file Supplementary_file_1.zip › Metabolomics sequencing data FC1.2/6.差异代谢物/heatmap-top50/横聚类/Exposure_Ctrl.pdf]

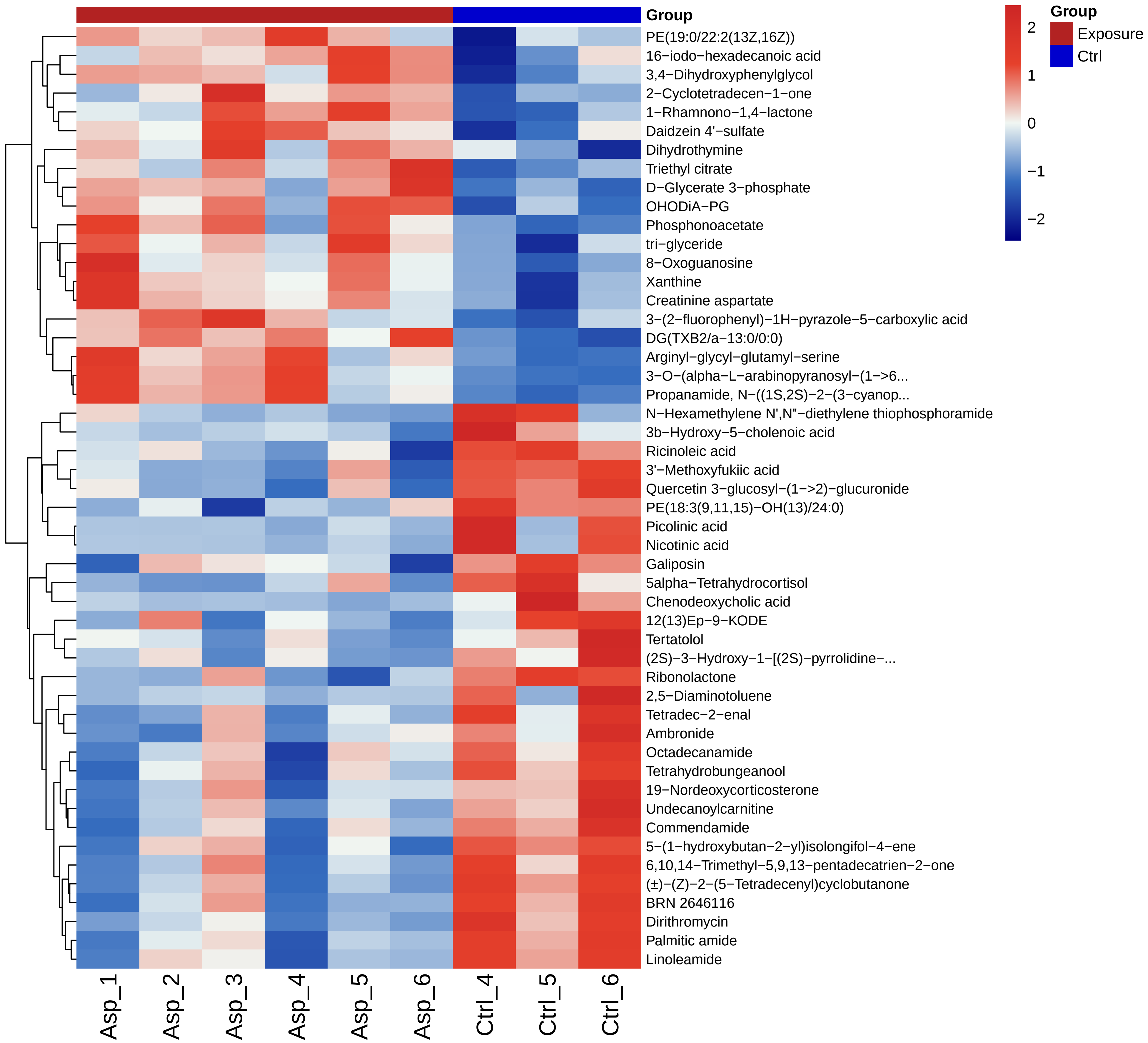

Group
Exposure
Ctrl
2
1
0
−1
−2
PE(19:0/22:2(13Z,16Z))
16−iodo−hexadecanoic acid
3,4−Dihydroxyphenylglycol
2−Cyclotetradecen−1−one
1−Rhamnono−1,4−lactone
Daidzein 4'−sulfate
Dihydrothymine
Triethyl citrate
D−Glycerate 3−phosphate
OHODiA−PG
Phosphonoacetate
tri−glyceride
8−Oxoguanosine
Xanthine
Creatinine aspartate
3−(2−fluorophenyl)−1H−pyrazole−5−carboxylic acid
DG(TXB2/a−13:0/0:0)
Arginyl−glycyl−glutamyl−serine
3−O−(alpha−L−arabinopyranosyl−(1−>6...
Propanamide, N−((1S,2S)−2−(3−cyanop...
N−Hexamethylene N',N''−diethylene thiophosphoramide
3b−Hydroxy−5−cholenoic acid
Ricinoleic acid
3'−Methoxyfukiic acid
Quercetin 3−glucosyl−(1−>2)−glucuronide
PE(18:3(9,11,15)−OH(13)/24:0)
Picolinic acid
Nicotinic acid
Galiposin
5alpha−Tetrahydrocortisol
Chenodeoxycholic acid
12(13)Ep−9−KODE
Tertatolol
(2S)−3−Hydroxy−1−[(2S)−pyrrolidine−...
Ribonolactone
2,5−Diaminotoluene
Tetradec−2−enal
Ambronide
Octadecanamide
Tetrahydrobungeanool
19−Nordeoxycorticosterone
Undecanoylcarnitine
Commendamide
5−(1−hydroxybutan−2−yl)isolongifol−4−ene
6,10,14−Trimethyl−5,9,13−pentadecatrien−2−one
(±)−(Z)−2−(5−Tetradecenyl)cyclobutanone
BRN 2646116
Dirithromycin
Palmitic amide
Linoleamide
Asp_1
Asp_2
Asp_3
Asp_4
Asp_5
Asp_6
Ctrl_4
Ctrl_5
Ctrl_6
